# Understanding why aspirin prevents cancer and why consuming very hot beverages and foods increases esophageal cancer risk. Controlling the division rates of stem cells is an important strategy to prevent cancer

**DOI:** 10.18632/oncoscience.257

**Published:** 2015-11-10

**Authors:** Miguel López-Lázaro

**Affiliations:** ^1^ Department of Pharmacology, Faculty of Pharmacy, University of Seville, Spain

**Keywords:** cancer prevention, carcinogenesis, chemoprevention, stem cell division theory of cancer, tissue injury and regeneration

## Abstract

Cancer is, in essence, a stem cell disease. The main biological cause of cancer is that stem cells acquire DNA alterations during cell division. The more stem cell divisions a tissue accumulates over a lifetime, the higher is the risk of cancer in that tissue. This explains why cancer is diagnosed millions of times more often in some tissues than in others, and why cancer incidence increases so dramatically with age. It may also explain why taking a daily low-dose aspirin for several years reduces the risk of developing and dying from cancer. Since aspirin use reduces PGE2 levels and PGE2 fuels stem cell proliferation, aspirin may prevent cancer by restricting the division rates of stem cells. The stem cell division model of cancer may also explain why regular consumption of very hot foods and beverages increases the risk of developing esophageal cancer. Given that tissue injury activates stem cell division for repair, the thermal injury associated with this dietary habit will increase esophageal cancer risk by inducing the accumulation of stem cell divisions in the esophagus. Using these two examples, here I propose that controlling the division rates of stem cells is an essential approach to preventing cancer.

## INTRODUCTION

Recent evidence indicates that we have cancer because our stem cells divide [1–3]. The division of stem cells is necessary to form, maintain and repair our tissues. But when they divide, their DNA gets damaged and our risk of cancer increases. Dividing cells are exposed, for example, to random mutations arising during DNA replication and to stochastic errors in the distribution of chromosomes between the daughter cells. Dividing cells are also exposed to a variety of damages caused by endogenous and environmental carcinogens. Importantly, stem cells represent the only cell population that can accumulate DNA damage during our whole life; they are the only tissue cells that can self-renew (copy themselves) and keep our DNA from the beginning of life until death. As we age, our tissues accumulate stem cell divisions, their stem cells accumulate DNA damage, and we accumulate cancer risk [1, 3]. This explains why most cancers are diagnosed in aged people [4].

Tomasetti and Vogelstein [2] recently found a highly positive correlation (Spearman's rho = 0.81; P < 3.5 × 10^−8^) between the number of normal stem cell divisions in a tissue and the risk of cancer in that tissue. The correlation applied to 31 cancer types and extended across five orders of magnitude [2]. This correlation means that if the total number of stem cell divisions in a tissue is 1, the risk of being diagnosed with cancer in that tissue is approximately 1X. If the number of stem cell divisions is 100, the risk is 100X. And if the total number of stem cell divisions in a tissue is 100,000, the risk of cancer in that tissue is approximately 100,000X [3]. In other words, the more stem cell divisions a tissue accumulates over a lifetime, the higher is the risk of developing cancer in that tissue. This implies that restricting the number of stem cell divisions will reduce the cancer risk, whereas promoting the division of stem cells will increase the cancer risk.

Here I discuss that aspirin use prevents cancer by restricting the division rates of stem cells, and that regular consumption of very hot beverages and foods raises the risk of esophageal cancer by increasing the number of stem cell divisions in the esophagus. Then, I propose that controlling the division rates of stem cells may be an essential approach to preventing cancer.

### Understanding why aspirin use reduces the risk of developing and dying from cancer

Taking a daily low-dose aspirin (75–100 mg) for several years reduces the risk of developing and dying from cancer [5–10]. A recent analysis of multiple clinical trials, cohort studies, and case-control studies indicates that aspirin use reduces cancer incidence by approximately 35% in colorectal cancer, 30% in esophageal and gastric cancers, 10% in breast and prostate cancers, and 5% in lung cancer. Aspirin use also reduces cancer mortality by approximately 50% in esophageal cancer, 40% in colorectal cancer, 35% in gastric cancer, 15% in lung and prostate cancers, and 5% in breast cancer [8]. In addition, aspirin reduces cancer mortality in patients previously diagnosed with specific tumors [11, 12]; this suggests that aspirin can prevent the development of established tumors in addition to reducing their occurrence.

Aspirin, a nonsteroidal anti-inflammatory drug (NSAID), exerts its known pharmacological properties through inhibition of constitutive (COX-1) and inducible (COX-2) cyclooxygenases. Inhibition of COX activity decreases the formation of prostanoids (e.g., PGE2 and TXA2) that promote inflammation, pain, fever, and platelet aggregation. Unlike other NSAIDs, aspirin causes an irreversible inhibition of the enzyme COX-1. The pharmacokinetic profile of aspirin after a low-dose administration (high concentrations in portal circulation followed by low concentrations in the general circulation) suggests that the activity of low-dose aspirin is mediated by irreversible inhibition of COX-1 activity in platelets (which are exposed to high concentrations of aspirin in the portal circulation). This in turn suggests that the antiplatelet action of aspirin may mediate its anticancer effects [10, 13]. However, aspirin-induced COX-1 inhibition in platelets lasts several days (platelets live several days and cannot synthesize new enzymes because they have no nucleus), and alternate-day aspirin does not result in considerable reductions in cancer risk [14, 15]. This suggests that the antiplatelet activity of aspirin cannot fully explain its anticancer effects. Low-dose aspirin may reduce the activity of COX-2 by inhibiting its synthesis rather than by direct inhibition of the enzyme. Aspirin and its stable metabolite salycilate can inhibit COX-2 gene transcription at lower concentrations (0.1 μM) [16] than those reached by both compounds in plasma (around 10 μM) after oral administration of low-dose aspirin [17]. This may explain why healthy volunteers taking 81 mg of aspirin for two weeks had a 45% reduction of PGE2 levels in urine samples [10].

The mechanism by which aspirin prevents cancer becomes obscure beyond COX inhibition [17, 18], probably because the main mechanism involved in cancer development has remained obscure until now. Tomasetti and Vogelstein [2] recently found a striking correlation between the number of normal stem cell divisions in a tissue and the risk of cancer in that tissue; this strongly suggests that the main biological cause of cancer is that stem cells acquire DNA damage during cell division. When normal stem cells accumulate sufficient damage, they become cancer stem cells (CSCs), which play a key role in tumor formation. Cell malignant transformation and tumor growth may therefore be prevented by restricting the proliferative activity of normal stem cells and CSCs [1, 3].

Prostaglandin E2 (PGE2) is known to activate normal stem cell proliferation to promote tissue regeneration [19, 20]. Enhanced PGE2 production is actually a universal response to tissue injury [19]. Through its interaction with Wnt, PGE2 plays a key role in stem cell self-renewal and tissue regeneration [19]. Recent evidence suggests that PGE2 may also activate CSC proliferation to promote tumor growth [21]. Following chemotherapy, an increase in this prostaglandin induced tumor repopulation by stimulating the division of CSCs; this effect was abrogated by a PGE2-neutralizing antibody and by the COX-2 inhibitor celecoxib [21].

By inhibiting COX-mediated PGE2 production, aspirin may restrict the proliferation of normal stem cells and CSCs. In other words, aspirin use probably lowers cancer risk because it prevents normal stem cells from becoming malignant. If they do, aspirin restricts their division rates and limits their tumor formation capacity.

### Understanding why regular consumption of very hot beverages and foods is an important risk factor for esophageal cancer

Globally, esophageal cancer is the ninth most common malignancy and the sixth most common cause of cancer-related death [22]. Smoking and heavy alcohol consumption are considered major risk factors for esophageal cancer [23, 24]. These risks factors, however, cannot explain the striking variations in esophageal cancer incidence among countries. For example, while esophageal cancer is the 23rd most common cancer in Greece and the 19th in Russia, it ranks second in Kenya and fourth in Iran [22]. Tobacco and alcohol use in Greece and Russia is however much more common than in Kenya and Iran [25] (Table 1). These data indicate that esophageal cancer is highly influenced by other risk factors. Identifying these factors is important for the development of cancer control strategies.

**Table 1:** Differences in esophageal cancer incidence and mortality among some countries are not explained by cigarette and alcohol use

Country	Incidence ranking^1^	Mortality ranking^1^	Cigarette use^2^	Alcohol use^3^
Greece	23	20	2,717	10.3
Russia	19	13	2,659	15.1
Kenya	2	1	415	4.3
Iran	4	2	925	1.0

1 Ranking of esophageal cancer with respect to other cancers in the country [22].

2 Mean cigarette consumption per capita in 2012 [25].

3 Mean alcohol consumption per capita (in liters of pure alcohol; 15+ years population) in 2010 (WHO Global status report on alcohol and health 2014).

Accumulating epidemiological evidence supports an association between regular consumption of high-temperature foods and drinks and an increased risk of esophageal cancer [26–28]. For example, most inhabitants of Golestan province, Iran, drink tea at temperatures higher than 60°C and in quantities greater than one liter per day [26]. A case-control study in this population showed that drinking hot tea and very hot tea was respectively associated with 2.07-fold and 8.16-fold increases in the risk of esophageal cancer compared with drinking lukewarm or warm tea [26]. A case-control study in the Rift Valley, Kenya, also found that drinking hot beverages was associated with a 12.78-fold increase in the risk of esophageal cancer [29]. Tobacco and alcohol use in this population was associated with 2.51-fold and 2.64-fold increases in the risk of this malignancy [29].

Despite this epidemiological evidence, regular consumption of high-temperature foods and drinks is not considered to be an important risk factor for esophageal cancer. This risk is mentioned in some cancer prevention guidelines and specialized web pages; however, it is not indicated that this dietary habit should be avoided, probably because the level of evidence is not considered adequate. In other guidelines and specialized websites, the cancer risk associated with this habit is not even mentioned. A possible reason is that the prevailing model of carcinogenesis states that cancer is caused by genetic mutations, and thermal tissue injury is not regarded as a significant source of mutations. But recent data indicate that tissue injury may be an important source of DNA damage to the cells that give rise to cancer [1].

When we drink or eat something hot enough to cause severe damage to the cells lining the esophagus, the stem cells of this tissue [30] have to divide to produce new cells to repair the damage. But when they divide, their DNA becomes exposed to unavoidable errors associated with cell division, and also becomes more vulnerable to the genotoxic activity of endogenous carcinogens (e.g., reactive oxygen and nitrogen species produced during inflammation) and exogenous carcinogens (e.g., tobacco and dietary carcinogens). The more times we ingest very hot foods and drinks, the more stem cell divisions will take place in the esophagus, the more DNA alterations will acquire the stem cells of this tissue, and the higher will be the risk of developing esophageal cancer.

In many countries, consuming very hot foods and drinks is probably less common than smoking or drinking alcohol. This means that tobacco and alcohol use may cause more cases of esophageal cancer in these countries and globally. However, the individual risk associated with this dietary habit may be comparable or even higher than that associated with tobacco and alcohol use. In other words, if an individual ingests scalding-hot beverages and foods regularly, his or her chance of developing esophageal cancer might be even higher than the risk of a smoker or a heavy alcohol drinker. The high incidence of esophageal cancer in countries where drinking scalding-hot tea is a common practice (e.g., Iran and Kenya) supports this possibility. Unfortunately, most people are unaware of the risk of cancer associated with this dietary habit.

There is sufficient epidemiological and mechanistic evidence to support that this dietary habit is an important risk factor for esophageal cancer. First, the incidence of esophageal cancer is generally very high in some countries where this dietary practice is common. Second, numerous case-control studies in different parts of the world show an increased risk of esophageal cancer in people with this habit [27]. Third, recent evidence indicates that the main biological cause of cancer is the accumulation of stem cell divisions in our tissues, and regular thermal injury to the esophagus will lead to the accumulation of stem cell divisions in this tissue.

Recognizing that regular consumption of high-temperature beverages and foods is an important risk factor for esophageal cancer is an essential step to take preventive measures. Taking preventive measures is crucial, because therapy is not usually curative even when this cancer is detected early; the five-year relative survival rates for esophageal cancer are about 20% [31]. In addition, since consuming very hot foods and drinks is avoidable and probably less addictive than smoking and drinking alcohol, these measures are likely to be effective. In individuals and populations with this dietary habit, simple measures like waiting for the soup to cool down or adding some cold water or milk to a boiling cup of tea may prevent more esophageal cancer deaths than therapy, and at a lower cost. This information should reach health policy-makers. It should reach people.

### Controlling the division rates of stem cells is an important strategy to prevent cancer

If the accumulation of DNA alterations in dividing stem cells is the major biological cause of cancer, controlling the division rates of stem cells may be an essential approach to preventing the disease. However, since stem cells have to divide to maintain and repair our tissues, one might think that restricting their division rates will compromise these important biological functions. One should be reminded that restricting an important biological function does not necessarily have to be harmful. For example, cholesterol plays an important role in maintaining the fluidity of cell membranes. Blood pressure is also necessary to ensure an adequate delivery of oxygen and nutrients to cells. However, restricting cholesterol and blood pressure levels does not necessarily have to be harmful. Avoiding hypercholesterolemia and hypertension through primary prevention (e.g., by limiting cholesterol and salt intake) and through secondary prevention (e.g., by using statins and ACE inhibitors) is actually an essential approach to preventing cardiovascular disease. Avoiding an overproliferation of stem cells may also be an essential approach to preventing cancer.

Primary cancer prevention can be achieved by identifying and controlling external factors that promote the division of stem cells. This research perspective shows that tissue injury is one of these factors. This can explain why the tissue injury associated with the consumption of very hot beverages and foods increases the risk of esophageal cancer [26, 27]. It may also explain why other types of tissue injury increase the risk of cancer [32–39]. For example, a case-control study found that women with breast carcinoma were more likely to report physical trauma to the breast in the previous five years than were women without breast carcinoma (OR = 3.3; 95% CI = 1.3–10.8; P < 0.0001) [39]. Another case-control study found that head trauma was associated with an increased risk of meningioma (OR = 1.83; 95% CI = 1.28- 2.62), especially head traumas occurring 10 to 19 years before diagnosis (OR = 4.33; 95% CI = 2.06-9.10) [38]. Epidemiological and mechanistic evidence indicates that tissue injury increases cancer risk; some types of tissue injury can be avoided.

Tissue injury activates signals, such as PGE2, that stimulate the division of stem cells to promote tissue regeneration. A recent article showed that inhibition of the prostaglandin-degrading enzyme 15-PGDH potentiates tissue regeneration by increasing tissue levels of PGE2 [20]. The authors discussed that 15-PGDH inhibition may be a valuable therapeutic strategy for tissue regeneration in diverse clinical contexts [20]. In my opinion, potentiating tissue regeneration may lead to an overproliferation of stem cells and may increase the risk of cancer. The benefit-risk profile of this therapeutic strategy should be carefully considered in each situation.

Introducing stem cells into our tissues for regenerative purposes may also increase our risk of cancer. The risk may be especially worrying when the cells have accumulated numerous divisions either in our body or as a result of their amplification *in vitro*. The tumorigenic potential of tissue stem cells derived from induced pluripotent stem cells (iPSCs) [40, 41] is probably higher than that of tissue stem cells derived from embryonic stem cells. It is already known that the methods for inducing pluripotency may introduce carcinogenic DNA alterations in the cells. It is also important to realize that the DNA of the cells used to generate iPSCs has been copied more times than the DNA of embryonic stem cells. Embryonic stem cells divide and produce tissue stem cells (also referred to as adult stem cells), which can self-renew during our whole life. At different stages in life, tissue stem cells produce progenitor cells, which proliferate to give rise to the differentiated cells (e.g., fibroblasts) generally used to generate iPSCs [40]. But in each of these cell divisions, our DNA has been copied and has accumulated alterations that may lead to cancer. The risk of cancer associated with stem cell therapies can be reduced by minimizing the number of cell divisions required to obtain the stem cells to be introduced into patients.

Cancer statistics support the intimate relationship between tissue regeneration and cancer risk. Although cancer incidence increases exponentially with age, a deceleration is usually observed late in life [4, 42]. According to SEER Cancer Statistics Review 1975– 2012, the risk of being diagnosed with cancer in our 80s is similar or even lower than in our 70s. Importantly, an overall decline in tissue regenerative potential also occurs in later years, which is attributed to a decline in stem cell functionality with age [43]. This suggests that a low tissue regenerative potential may protect us against cancer, and that potentiating tissue regeneration may facilitate cancer development. The risk of cancer associated with regenerative therapies that promote the division of endogenous stem cells, or that employ exogenous stem cells, should be carefully considered in each situation.

Knowing that the division rates of stem cells can be controlled and altered by numerous factors (e.g., mechanical, physical, chemical and neural) [44] will help identify new environmental carcinogens. Future research might show that a variety of non-genotoxic chemicals generally recognized as safe can raise cancer risk by increasing the division rates of stem cells. Future research may also consistently show that regular exposure to electromagnetic fields (EMFs) increases cancer risk. Evidence has accumulated that radiofrequency EMFs (emitted by mobile phones and Wi-Fi routers, for example) increase the risk of cancer [45–47]. Recently, many scientists have expressed concern about the long-term health effects associated with the ubiquitous and increasing exposure to EMFs generated by electric and wireless devices (https://emfscientist.org/). However, no mechanism by which EMFs could cause cancer has been established. Unlike ionizing radiation, EMFs are low-energy radiations that cannot cause apparent damage to DNA or cells. This may prevent health organizations from adopting more protective measures against this rapidly growing form of environmental pollution worldwide. In my opinion, EMFs may raise cancer risk by increasing the division rates of stem cells [44, 48–50]. They might do so, for example, by disrupting the electrical interactions between stem cells and their niches. It is important to realize that the risk of cancer is not only increased by agents that directly or indirectly interact with the DNA, but also by those promoting the division of stem cells [1]. Promoting the division of stem cells will result in DNA alterations associated with cell division (e.g., mutation arising during DNA replication), which will occur even in the absence of any DNA damaging agent.

The division rates of stem cells can also be controlled by identifying physiological signals involved in the regulation of their division. This is important to reduce the risk of cancer associated with external factors not yet identified or that are difficult to control. For example, although we cannot prevent some types of tissue injury, we may control mediators (e.g., PGE2) that stimulate the division of stem cells following tissue injury. Controlling these mediators (e.g., with aspirin) may prevent the possible stem cell overproliferation associated with wound overhealing and may therefore reduce cancer risk [33, 35, 36]. The division rates of stem cells are probably influenced by a variety of cytokines, growth factors and hormones. Identifying and controlling these chemical signals may be crucial for preventing cancer. This research perspective has discussed that restricting the levels of the cytokine PGE2 by taking a daily low-dose aspirin may be an effective approach to reducing cancer incidence and mortality. Restricting the levels of the growth hormone (GH) and the insulin-like growth factor 1 (IGF1), the major mediator of the effects of GH, may also restrict the division rates of stem cells and may reduce cancer risk [51–55]. Deficiency in GH receptor and congenital IGF1 deficiency are known to confer protection against cancer [51–53]. High serum concentrations of IGF1 are associated with an increased risk of several cancers, including breast, prostate, colorectal, and lung cancers [54, 55]. In addition, IGF1 promotes the division of normal stem cells and CSCs [56–61]. Controlling the levels of GH and IGF1 may therefore be an important strategy to prevent cancer. Interestingly, fasting and low protein intake can reduce serum IGF1 levels, and can also reduce tumor growth in animals and cancer risk in some human populations [62, 63]. Restriction of particular nutrients, such as essential amino acids, may also restrict the division rates of stem cells [64–66]. When the levels of particular nutrients are scarce, stem cells may somehow sense that it is not a good time for division. Identifying and restricting these nutrients may be an important strategy to control the division rates of stem cells and thus prevent cancer.

## CONCLUDING REMARKS

There is a conflict between the message of this research perspective and that of the article by Tomasetti and Vogelstein [2]. I propose that the major biological cause of cancer is preventable, whereas they suggested that it is not. As mentioned before, they found a highly positive correlation between the number of stem cell divisions in a tissue and the risk of cancer in that tissue (Spearman's rho = 0.81; P < 3.5 × 10^−8^). Pearson's linear correlation 0.804 was also highly significant (P < 5.15 × 10^−8^). They discussed that a linear correlation equal to 0.804 indicated that 65% of the differences in cancer risk among tissues could be explained by the number of stem cell divisions in those tissues. Then, they interpreted that the parameters “stem cell divisions” and “DNA replication mutations” could be interchanged, and proposed that approximately two-thirds (65%) of the mutations required to originate a cancer were random mutations arising during DNA replication. The other third were mutations resulting from hereditary or environmental factors. Since we cannot prevent DNA polymerases from making random mistakes when they replicate our DNA, they proposed that the major cause of cancer could not be prevented [2].

The striking differences in esophageal cancer incidence among countries (Table 1) are difficult to explain if the major biological cause of this cancer is random [2]. The marked reductions in esophageal cancer risk associated with aspirin use [8] are also difficult to explain if the major biological cause of this cancer is not preventable [2]. The raw data provided by Tomasetti and Vogelstein [2] show a strong association between the number of stem cell divisions and cancer incidence. This probably means that the major cause of cancer is that stem cells acquire DNA alterations during cell division, rather than that stem cells acquire random mutations during DNA replication. In my opinion, the parameters “stem cell divisions” and “DNA replication mutations” are not interchangeable. The main reason is that the mutations arising during DNA replication are random and unavoidable, while the division of stem cells is not a random and unavoidable process. The division of stem cells is highly influenced by external factors (e.g., tissue injury) and physiological signals (e.g., PGE2). Controlling these factors and signals will limit the division rates of stem cells; this will restrict the acquisition of DNA alterations during cell division, including those arising during DNA replication.

As discussed by Tomasetti and Vogelstein, our stem cells will acquire DNA alterations no matter what we do. Stem cells have to divide, and some errors arising during cell division are unavoidable. Cancer prevention will partially protect stem cells from getting damaged and will lead to a cancer-free life in many cases; this partial protection may be sufficient to avoid in many cases “the straw that breaks the camel's back”. In other cases, primary prevention efforts will not stop some stem cells from becoming malignant. But prevention is still possible in these cases. The accumulation of DNA alterations in stem cells can make them vulnerable to specific pharmacological and non-pharmacological interventions. Finding a chemopreventive drug to selectively kill mutated stem cells before they give rise to cancer is possible. Mutated stem cells may also be eliminated by selective restriction of specific amino acids [67]. In my opinion, all cancer cases are potentially preventable. Future research will hopefully show the way to achieve it. In the meantime, controlling the division rates of stem cells may be an essential strategy to prevent cancer. This can be accomplished by identifying and controlling external factors that promote the division of stem cells, and by identifying and controlling internal signals that regulate their division rates.

## References

[1] Lopez-Lazaro M (2015). Stem cell division theory of cancer. Cell Cycle.

[2] Tomasetti C, Vogelstein B (2015). Cancer etiology. Variation in cancer risk among tissues can be explained by the number of stem cell divisions. Science.

[3] Lopez-Lazaro M (2015). The migration ability of stem cells can explain the existence of cancer of unknown primary site. Rethinking metastasis. Oncoscience.

[4] DePinho RA (2000). The age of cancer. Nature.

[5] Rothwell PM, Fowkes FG, Belch JF, Ogawa H, Warlow CP, Meade TW (2011). Effect of daily aspirin on long-term risk of death due to cancer: analysis of individual patient data from randomised trials. Lancet.

[6] Rothwell PM, Wilson M, Price JF, Belch JF, Meade TW, Mehta Z (2012). Effect of daily aspirin on risk of cancer metastasis: a study of incident cancers during randomised controlled trials. Lancet.

[7] Rothwell PM, Price JF, Fowkes FG, Zanchetti A, Roncaglioni MC, Tognoni G, Lee R, Belch JF, Wilson M, Mehta Z, Meade TW (2012). Short-term effects of daily aspirin on cancer incidence, mortality, and non-vascular death: analysis of the time course of risks and benefits in 51 randomised controlled trials. Lancet.

[8] Cuzick J, Thorat MA, Bosetti C, Brown PH, Burn J, Cook NR, Ford LG, Jacobs EJ, Jankowski JA, La Vecchia C, Law M, Meyskens F, Rothwell PM (2015). Estimates of benefits and harms of prophylactic use of aspirin in the general population. Ann. Oncol.

[9] Torjesen I (2014). Daily aspirin reduces risk of developing and dying from cancer, researchers find. BMJ.

[10] Sinha G (2015). More evidence that aspirin lowers cancer risk. J. Natl Cancer Inst.

[11] Chan AT, Ogino S, Fuchs CS (2009). Aspirin use and survival after diagnosis of colorectal cancer. JAMA.

[12] Jacobs EJ, Newton CC, Stevens VL, Campbell PT, Freedland SJ, Gapstur SM (2014). Daily aspirin use and prostate cancer-specific mortality in a large cohort of men with nonmetastatic prostate cancer. J Clin Oncol.

[13] Thun MJ, Jacobs EJ, Patrono C (2012). The role of aspirin in cancer prevention. Nat Rev Clin Oncol.

[14] Cook NR, Lee IM, Gaziano JM, Gordon D, Ridker PM, Manson JE, Hennekens CH, Buring JE (2005). Low-dose aspirin in the primary prevention of cancer: the Women's Health Study: a randomized controlled trial. JAMA.

[15] Cook NR, Lee IM, Zhang SM, Moorthy MV, Buring JE (2013). Alternate-day, low-dose aspirin and cancer risk: long-term observational follow-up of a randomized trial. Ann Intern Med.

[16] Xu XM, Sansores-Garcia L, Chen XM, Matijevic-Aleksic N, Du M, Wu KK (1999). Suppression of inducible cyclooxygenase 2 gene transcription by aspirin and sodium salicylate. Proc Natl Acad Sci. U.S.A.

[17] Alfonso L, Ai G, Spitale RC, Bhat GJ (2014). Molecular targets of aspirin and cancer prevention. Br J Cancer.

[18] Usman MW, Luo F, Cheng H, Zhao JJ, Liu P (2015). Chemopreventive effects of aspirin at a glance. Biochim Biophys Acta.

[19] Goessling W, North TE, Loewer S, Lord AM, Lee S, Stoick-Cooper CL, Weidinger G, Puder M, Daley GQ, Moon RT, Zon LI (2009). Genetic interaction of PGE2 and Wnt signaling regulates developmental specification of stem cells and regeneration. Cell.

[20] Zhang Y, Desai A, Yang SY, Bae KB, Antczak MI, Fink SP, Tiwari S, Willis JE, Williams NS, Dawson DM, Wald D, Chen WD, Wang Z (2015). Tissue regeneration. Inhibition of the prostaglandin-degrading enzyme 15-PGDH potentiates tissue regeneration. Science.

[21] Kurtova AV, Xiao J, Mo Q, Pazhanisamy S, Krasnow R, Lerner SP, Chen F, Roh TT, Lay E, Ho PL, Chan KS (2015). Blocking PGE2-induced tumour repopulation abrogates bladder cancer chemoresistance. Nature.

[22] Fitzmaurice C, Dicker D, Pain A, Hamavid H, Moradi-Lakeh M, MacIntyre MF, Allen C, Hansen G, Woodbrook R, Wolfe C, Hamadeh RR, Moore A, Werdecker A (2015). The Global Burden of Cancer 2013. JAMA Oncol.

[23] Castellsague X, Munoz N, De Stefani E, Victora CG, Castelletto R, Rolon PA, Quintana MJ (1999). Independent and joint effects of tobacco smoking and alcohol drinking on the risk of esophageal cancer in men and women. Int J Cancer.

[24] Freedman ND, Abnet CC, Leitzmann MF, Mouw T, Subar AF, Hollenbeck AR, Schatzkin A (2007). A prospective study of tobacco, alcohol, and the risk of esophageal and gastric cancer subtypes. Am J Epidemiol.

[25] Ng M, Freeman MK, Fleming TD, Robinson M, Dwyer-Lindgren L, Thomson B, Wollum A, Sanman E, Wulf S, Lopez AD, Murray CJ, Gakidou E (2014). Smoking prevalence and cigarette consumption in 187 countries, 1980-2012. JAMA.

[26] Islami F, Pourshams A, Nasrollahzadeh D, Kamangar F, Fahimi S, Shakeri R, Abedi-Ardekani B, Merat S, Vahedi H, Semnani S, Abnet CC, Brennan P, Moller H (2009). Tea drinking habits and oesophageal cancer in a high risk area in northern Iran: population based case-control study. BMJ.

[27] Islami F, Boffetta P, Ren JS, Pedoeim L, Khatib D, Kamangar F (2009). High-temperature beverages and foods and esophageal cancer risk—a systematic review. Int J Cancer.

[28] Chen Y, Tong Y, Yang C, Gan Y, Sun H, Bi H, Cao S, Yin X, Lu Z (2015). Consumption of hot beverages and foods and the risk of esophageal cancer: a meta-analysis of observational studies. BMC Cancer.

[29] Patel K, Wakhisi J, Mining S, Mwangi A, Patel R (2013). Esophageal Cancer, the Topmost Cancer at MTRH in the Rift Valley, Kenya, and Its Potential Risk Factors. ISRN Oncol.

[30] DeWard AD, Cramer J, Lagasse E (2014). Cellular heterogeneity in the mouse esophagus implicates the presence of a nonquiescent epithelial stem cell population. Cell Rep.

[31] Siegel RL, Miller KD, Jemal A (2015). Cancer statistics, 2015. CA Cancer J Clin.

[32] Grasso P, Sharratt M, Cohen AJ (1991). Role of persistent, non-genotoxic tissue damage in rodent cancer and relevance to humans. Annu Rev Pharmacol Toxicol.

[33] Dunham LJ (1972). Cancer in man at site of prior benign lesion of skin or mucous membrane: a review. Cancer Res.

[34] Dolberg DS, Hollingsworth R, Hertle M, Bissell MJ (1985). Wounding and its role in RSV-mediated tumor formation. Science.

[35] Dvorak HF (1986). Tumors: wounds that do not heal. Similarities between tumor stroma generation and wound healing. N. Engl J Med.

[36] Schafer M, Werner S (2008). Cancer as an overhealing wound: an old hypothesis revisited. Nat Rev Mol Cell Biol.

[37] Kuraishy A, Karin M, Grivennikov SI (2011). Tumor promotion via injury- and death-induced inflammation. Immunity.

[38] Phillips LE, Koepsell TD, van Belle G, Kukull WA, Gehrels JA, Longstreth WT (2002). History of head trauma and risk of intracranial meningioma: population-based case-control study. Neurology.

[39] Rigby JE, Morris JA, Lavelle J, Stewart M, Gatrell AC (2002). Can physical trauma cause breast cancer?. Eur J Cancer Prev.

[40] Takahashi K, Tanabe K, Ohnuki M, Narita M, Ichisaka T, Tomoda K, Yamanaka S (2007). Induction of pluripotent stem cells from adult human fibroblasts by defined factors. Cell.

[41] Wilson KD, Wu JC (2015). Induced pluripotent stem cells. JAMA.

[42] Frank SA (2004). Age-specific acceleration of cancer. Curr Biol.

[43] Rando TA (2006). Stem cells, ageing and the quest for immortality. Nature.

[44] Scadden DT (2006). The stem-cell niche as an entity of action. Nature.

[45] Hardell L, Carlberg M (2015). Mobile phone and cordless phone use and the risk for glioma - Analysis of pooled case-control studies in Sweden, 1997-2003 and 2007-2009. Pathophysiology.

[46] Morgan LL, Miller AB, Sasco A, Davis DL (2015). Mobile phone radiation causes brain tumors and should be classified as a probable human carcinogen (2A) (review). Int J Oncol.

[47] Lerchl A, Klose M, Grote K, Wilhelm AF, Spathmann O, Fiedler T, Streckert J, Hansen V, Clemens M (2015). Tumor promotion by exposure to radiofrequency electromagnetic fields below exposure limits for humans. Biochem Biophys. Res. Commun.

[48] Blackiston DJ, McLaughlin KA, Levin M (2009). Bioelectric controls of cell proliferation: ion channels, membrane voltage and the cell cycle. Cell Cycle.

[49] Levin M (2014). Molecular bioelectricity: how endogenous voltage potentials control cell behavior and instruct pattern regulation *in vivo*. Mol Biol Cell.

[50] Ross CL, Siriwardane M, Almeida-Porada G, Porada CD, Brink P, Christ GJ, Harrison BS (2015). The effect of low-frequency electromagnetic field on human bone marrow stem/progenitor cell differentiation. Stem Cell Res.

[51] Guevara-Aguirre J, Balasubramanian P, Guevara-Aguirre M, Wei M, Madia F, Cheng CW, Hwang D, Martin-Montalvo A, Saavedra J, Ingles S, de Cabo R, Cohen P, Longo VD (2011). Growth hormone receptor deficiency is associated with a major reduction in pro-aging signaling, cancer, and diabetes in humans. Sci Transl Med.

[52] Steuerman R, Shevah O, Laron Z (2011). Congenital IGF1 deficiency tends to confer protection against post-natal development of malignancies. Eur J Endocrinol.

[53] Melnik BC, John SM, Schmitz G (2011). Over-stimulation of insulin/IGF-1 signaling by western diet may promote diseases of civilization: lessons learnt from laron syndrome. Nutr Metab (Lond).

[54] Furstenberger G, Senn HJ (2002). Insulin-like growth factors and cancer. Lancet Oncol.

[55] Renehan AG, Zwahlen M, Minder C, O'Dwyer ST, Shalet SM, Egger M (2004). Insulin-like growth factor (IGF)-I, IGF binding protein-3, and cancer risk: systematic review and meta-regression analysis. Lancet.

[56] Wang L, Schulz TC, Sherrer ES, Dauphin DS, Shin S, Nelson AM, Ware CB, Zhan M, Song CZ, Chen X, Brimble SN, McLean A, Galeano MJ (2007). Self-renewal of human embryonic stem cells requires insulin-like growth factor-1 receptor and ERBB2 receptor signaling. Blood.

[57] Baik I, Devito WJ, Ballen K, Becker PS, Okulicz W, Liu Q, Delpapa E, Lagiou P, Sturgeon S, Trichopoulos D, Quesenberry PJ, Hsieh CC (2005). Association of fetal hormone levels with stem cell potential: evidence for early life roots of human cancer. Cancer Res.

[58] Dallas NA, Xia L, Fan F, Gray MJ, Gaur P, van Buren G, Samuel S, Kim MP, Lim SJ, Ellis LM (2009). Chemoresistant colorectal cancer cells, the cancer stem cell phenotype, and increased sensitivity to insulin-like growth factor-I receptor inhibition. Cancer Res.

[59] Hart LS, Dolloff NG, Dicker DT, Koumenis C, Christensen JG, Grimberg A, El Deiry WS (2011). Human colon cancer stem cells are enriched by insulin-like growth factor-1 and are sensitive to figitumumab. Cell Cycle.

[60] Murakami A, Takahashi F, Nurwidya F, Kobayashi I, Minakata K, Hashimoto M, Nara T, Kato M, Tajima K, Shimada N, Iwakami S, Moriyama M, Moriyama H (2014). Hypoxia increases gefitinib-resistant lung cancer stem cells through the activation of insulin-like growth factor 1 receptor. PLoS One.

[61] Bu Y, Jia QA, Ren ZG, Zhang JB, Jiang XM, Liang L, Xue TC, Zhang QB, Wang YH, Zhang L, Xie XY, Tang ZY (2014). Maintenance of stemness in oxaliplatin-resistant hepatocellular carcinoma is associated with increased autocrine of IGF1. PLoS One.

[62] Lee C, Raffaghello L, Brandhorst S, Safdie FM, Bianchi G, Martin-Montalvo A, Pistoia V, Wei M, Hwang S, Merlino A, Emionite L, de Cabo R, Longo VD (2012). Fasting cycles retard growth of tumors and sensitize a range of cancer cell types to chemotherapy. Sci Transl Med.

[63] Levine ME, Suarez JA, Brandhorst S, Balasubramanian P, Cheng CW, Madia F, Fontana L, Mirisola MG, Guevara-Aguirre J, Wan J, Passarino G, Kennedy BK, Wei M (2014). Low protein intake is associated with a major reduction in IGF-1, cancer, and overall mortality in the 65 and younger but not older population. Cell Metab.

[64] Miller RA, Buehner G, Chang Y, Harper JM, Sigler R, Smith-Wheelock M (2005). Methionine-deficient diet extends mouse lifespan, slows immune and lens aging, alters glucose, T4, IGF-I and insulin levels, and increases hepatocyte MIF levels and stress resistance. Aging Cell.

[65] Cavuoto P, Fenech MF (2012). A review of methionine dependency and the role of methionine restriction in cancer growth control and life-span extension. Cancer Treat Rev.

[66] Lamb R, Harrison H, Smith DL, Townsend PA, Jackson T, Ozsvari B, Martinez-Outschoorn UE, Pestell RG, Howell A, Lisanti MP, Sotgia F (2015). Targeting tumor-initiating cells: Eliminating anabolic cancer stem cells with inhibitors of protein synthesis or by mimicking caloric restriction. Oncotarget.

[67] Lopez-Lazaro M (2015). Selective amino acid restriction therapy (SAART): a non-pharmacological strategy against all types of cancer cells. Oncoscience.

